# Supplementary material for: Antilisterial Properties of Selected Strains from the Autochthonous Microbiota of a Swiss Artisan Soft Smear Cheese
Source: Foods. 2024 Oct 30;13(21):3473. doi: 10.3390/foods13213473 (PMC11545730; doi:10.3390/foods13213473)
Supplement: Supplementary file 1 [file foods-13-03473-s001.zip › Figure_S2_undescribed_species.pdf]

	FAM 20857	FAM 20858	FAM 24228	FAM 26254	FAM 26255	FAM 24227	INB8	DSM 104272
FAM 20857	*	99.2	98.74	99.03	96.82	96.31	95.05	94.24
FAM 20858	98.78	*	98.61	99.04	96.52	96.15	94.7	94.03
FAM 24228	98.62	98.82	*	98.87	96.69	95.97	94.97	94.14
FAM 26254	98.86	99.3	98.82	*	96.78	96.26	94.98	94.11
FAM 26255	96.76	96.76	96.7	96.68	*	96.73	93.93	95.33
FAM 24227	96.16	96.29	95.95	96.21	96.54	*	93.85	95.27
INB8	95.09	95.07	95.17	94.96	93.97	93.91	*	93.14
DSM 104272	93.76	93.9	93.81	93.78	94.77	94.94	92.75	*

Figure S2a Average Nucleotide Identity based on Blast+ (**ANIb**) values for 6 strains of ***Ruoffia* sp.**, *Ruoffia halotolerans* INB8, and *Ruoffia tabacinasalis* DSM 104272 calculated according to [44] (https://jspecies.ribohost.com/jspeciesws/).

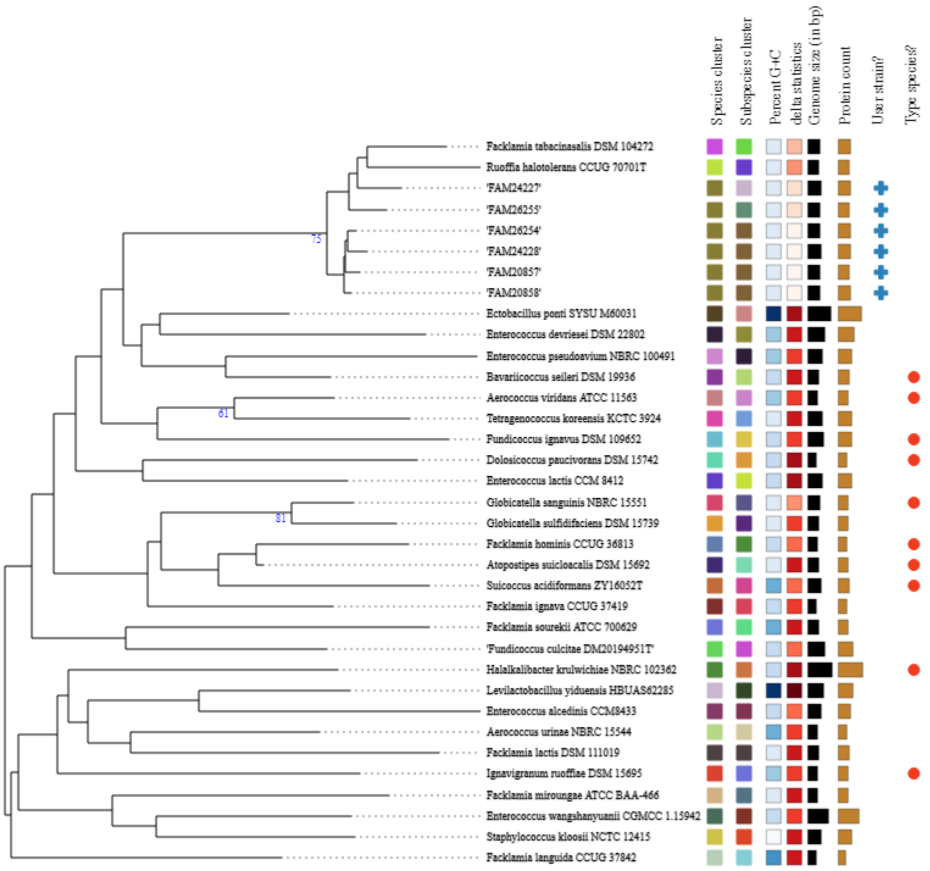

Figure S2b Genome Blast Distance Phylogeny GBDP tree (whole-genome sequence-based) for 6 strains of ***Ruoffia* sp.** as calculated on the Type Strain Genome Server (https://tygs.dsmz.de/; [45]) on January 11, 2024.

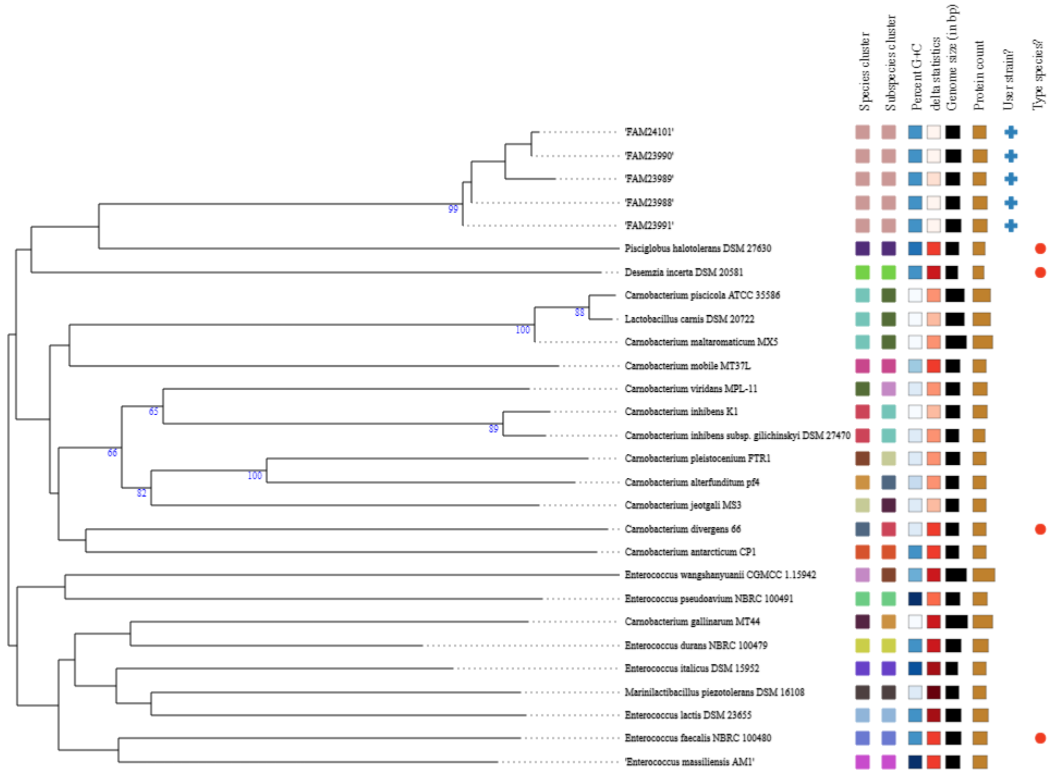

Figure S2c Genome Blast Distance Phylogeny GBDP tree (whole-genome sequence-based) for 5 strains of ***Desemzia* sp.** as calculated on the Type Strain Genome Server (https://tygs.dsmz.de/; [45]) on January 11, 2024.

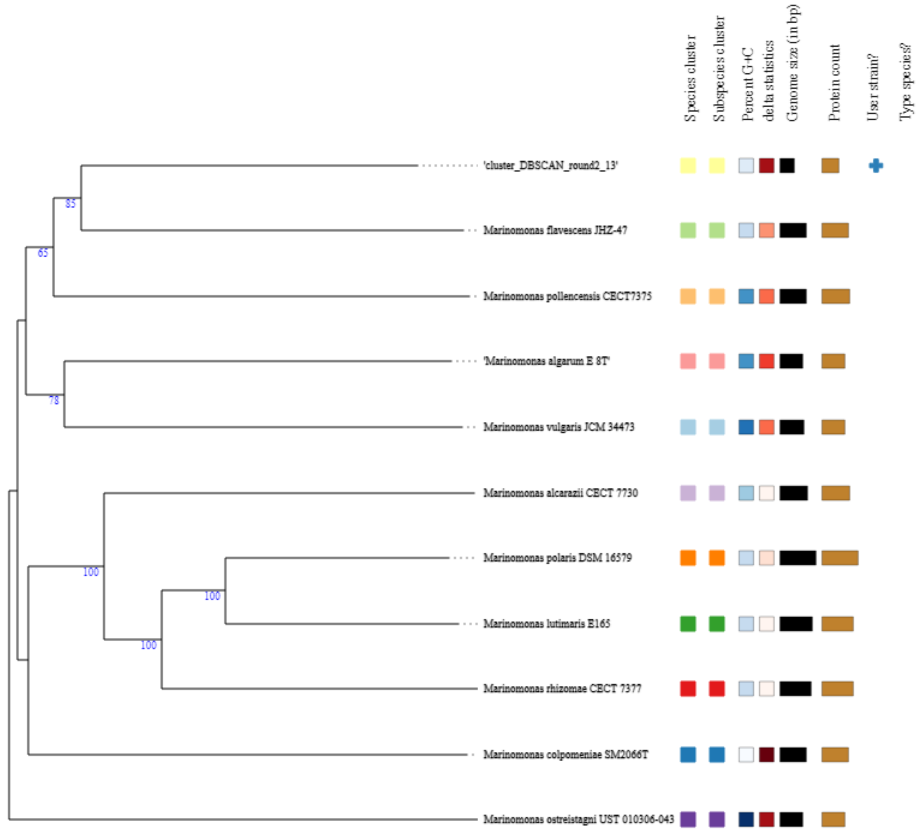

Figure S2d Genome Blast Distance Phylogeny GBDP tree (whole-genome sequence-based) for ***Marinomonas* sp.** (MAG DBSCAN_round2_13, 35 days-old rind of VMO, Dairy D, trial I, replicate 1) as calculated on the Type Strain Genome Server (https://tygs.dsmz.de/; [45]) on January 11, 2024.

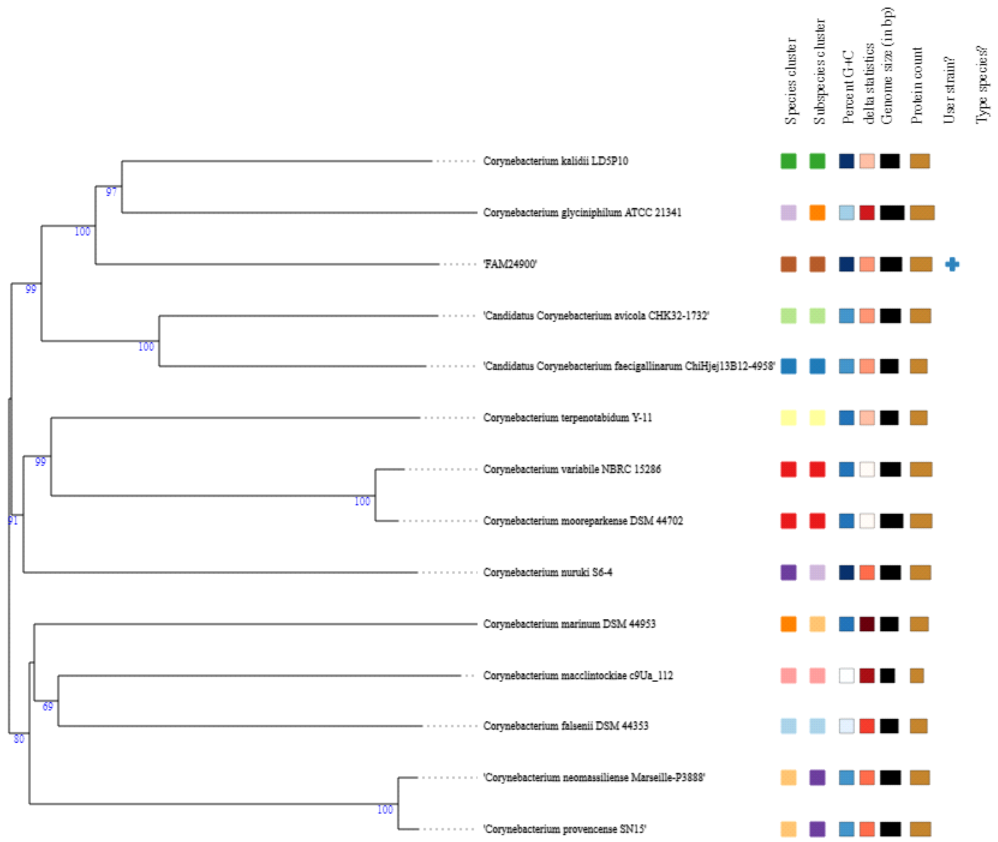

Figure S2e Genome Blast Distance Phylogeny GBDP tree (whole-genome sequence-based) for ***Corynebacterium* sp.** FAM24900 as calculated on the Type Strain Genome Server (https://tygs.dsmz.de/; [45]) on January 11, 2024.

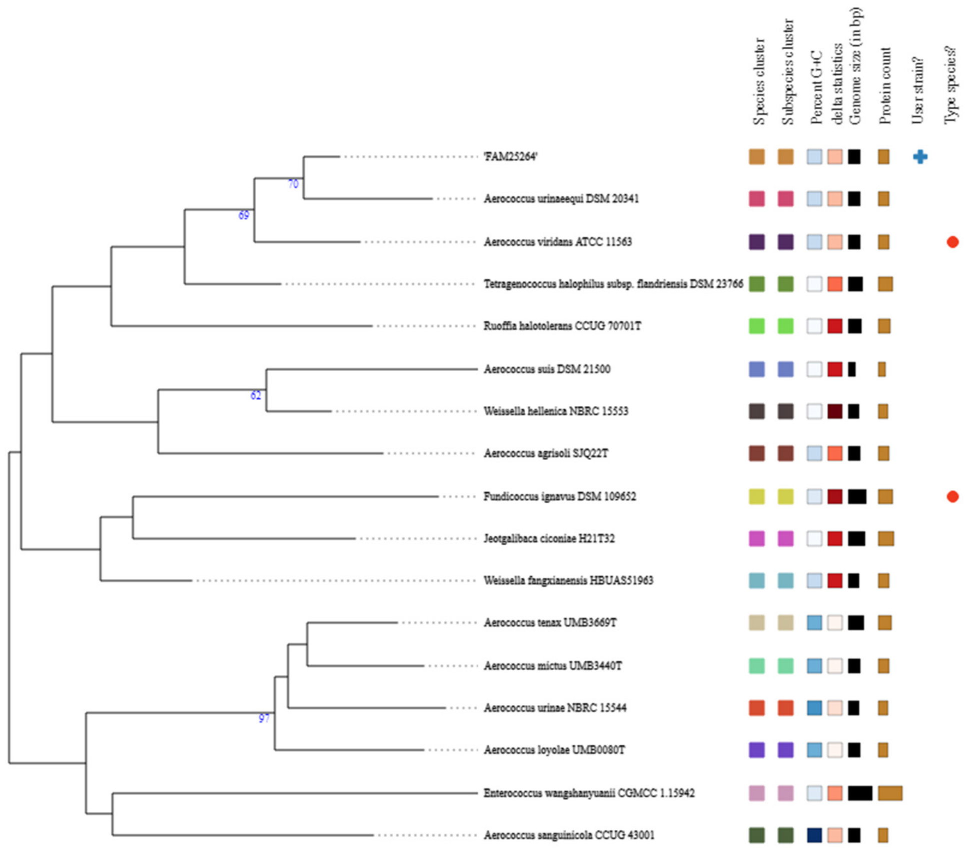

Figure S2f Genome Blast Distance Phylogeny GBDP tree (whole-genome sequence-based) for ***Aerococcus* sp.** FAM25264 as calculated on the Type Strain Genome Server (https://tygs.dsmz.de/; [45]) on January 11, 2024.
