# Supplementary material for: Antilisterial Properties of Selected Strains from the Autochthonous Microbiota of a Swiss Artisan Soft Smear Cheese
Source: Foods. 2024 Oct 30;13(21):3473. doi: 10.3390/foods13213473 (PMC11545730; doi:10.3390/foods13213473)
Supplement: Supplementary file 1 [file foods-13-03473-s001.zip › File_S1_method_real_time_PCR.pdf]

Primers and probe design for *Marinilactibacillus psychrotolerans*

A 77-bp fragment with the primers MapsSodA_fw (5'- CCA CAT ATC GAT GAG GAG ACT ATG C -3'), MapsSodA_rev (5'- GCA GCG TTC ACT TTA GTC ACG TA -3') and Taqman®probe (6'FAM- CCA CCA CGA TAA GCA CCA TGC TGC -TAMRA-3') was designed on the *sodA* gene which codes for the manganese-dependent superoxide dismutase protein (GenBank: VBTE01000051.1, Region 5137-5213) by using the Primer Express® software (version 3.0.1, Applied Biosystems, USA). Primers and probe were purchased from Microsynth (Balgach, Switzerland).

Primers and probe design for *Desemzia* sp.

A 68-bp fragment with the primers D.inc_fw (5'- CGC AAA CCA CAG CTT CTT CTG -3'), D.inc_rev (5'- TCA ACG CTC CTG TTG GTT CA -3') and Taqman® MGB (minor groove binding) probe (6'FAM- ATT TTA TCT CCA AAT GGC G -MGBQ5-3') was designed on the *sodA* gene which codes for the manganese-dependent superoxide dismutase protein (GenBank: JBGQQU000000002.1, Region 39142-39209) by using the Primer Express® software (version 3.0.1, Applied Biosystems, USA). Primers and probe were purchased from Microsynth (Switzerland).

Primers and probe design for *Ruoffia sp.*

A 68-bp fragment with the primers F.tab_2_fw (5'- GGA AAT CCA TCA CGG CAA AC -3'), F.tab_2_rev (5'- CAG TTC CTT CCA ATG CAT CAT TTA -3') and Taqman® MGB (minor groove binding) probe (6'FAM-CCA TAA TGG ATA TGT AAC GAA A -MGBQ5-3') was designed on the *sodA* gene which codes for the manganese-dependent superoxide dismutase protein (GenBank: VBSP01000008.1, Region 6914-6847) by using the Primer Express® software (version 3.0.1, Applied Biosystems, USA). Primers and probe were purchased from Microsynth (Switzerland).

Primers and probe design for *mesY* from *Leuconostoc mesenteroides*

A 66-bp fragment with the primers qMesY_F (5'- GGC AGC TTC TCC CCA GTT AA -3'), qMesY_R (5'- AAG TAT TAT GGG AAT GGT GTT CAC TGT -3') and Taqman® MGB (minor groove binding) probe (6'FAM- AGA GCA TCC ACT TTT T -MGBQ5-3') was designed on the mesentericin Y105 precursor (GenBank AY286003.1, Region 429-494) by using the Primer Express® software (version 3.0.1, Applied Biosystems, USA). Primers and probe were purchased from Microsynth (Switzerland).

Real-time PCR conditions

The Takyon™ No Rox 2x MasterMix UNG (Eurogentec, Belgium) was used for all qPCR measurements. The real-time PCR was carried out in a final reaction volume of 12 µl containing 0.3 µM of each primer, 0.1 µM probe, 2 x reaction buffer and 2 µl of DNA. Amplifications were run in a Corbett Rotor-Gene 6000 (Qiagen, Germany) (Marini, Desemzia and Ruoffia) and in a Corbett Rotor-Gene 3000 (MesY) using the following program: 2 min at 50 °C, 3 min at 95 °C, and 40 cycles of 3 s at 95 °C and 20 s at 60 °C. All real-time data were analyzed using the Rotor-Gene software version 1.7.87 (Qiagen) using a threshold of 0.03 for the quantification cycle (Cq) value determination. Each sample was measured in duplicate and non-template controls tested negative in all PCR runs.

Standard curves for quantification

Plasmids for standards curves were constructed by inserting a part of the *sodA* gene resp. *MesY* gene into a pGEM®-t easy Vector System I (Promega, Switzerland) according the procedure described in [48].

Marinilactibacillus psychrotolerans

A 290 bp PCR product was amplified with the primers MapsClo_fw: (5'-ACC TGA ATT ACC TTA TGC TT-3') and MapsClo_rev (5'- CTA ATT CTC CAG AAG GTT TC-3') from the

gDNA of *Marinilactibacillus psychrotolerans* DSM19582 in a 2720 Thermal cycler (Life Technologies, United States) with the following program: 95°C for 10 min, then 40 cycles of 95°C 30 s, 51°C 30 s, 72°C 30 s and a final amplification of 7 min at 72°C.

Desemzia sp.

A 461 bp PCR product was amplified with the primers D.inc_clo_fw: (5-ACA CCC TGA ATT AGC GGA CA-3') and D.inc_clo_rev (5'-CG TAA CGT TTG CCT GCT TCT-3') from the gDNA of *Desemzia sp.* FAM24101 in a 2720 Thermal cycler (Life Technologies, United States) with the following program: 95°C for 10 min, then 40 cycles of 95°C 30 s, 59°C 30 s, 72°C 30 s and a final amplification of 7 min at 72°C.

Ruoffia sp.

A 498 bp PCR product was amplified with the primers F.tab_clo_fw (5'-AGC CAG TTA TCG ATA AGG AAA CA-3') and F.tab_clo_rev (5'-AGT ATT CTG GAC GAC GGT TTT G-3') from the gDNA of *Ruoffia sp.* FAM24227 in a 2720 Thermal cycler (Life Technologies, United States) with the following program: 95°C for 10 min, then 40 cycles of 95°C 30 s, 58°C 30 s, 72°C 30 s and a final amplification of 7 min at 72°C.

MesY **from** ***Leuconostoc mesenteroides***

A 169 bp PCR product was amplified with the primers MesY_fw (5'-ACC AAA ATC CAT TTC CAC CA-3') and MesY_rev (5'-TCT GTG GAA GCA TAT CAG CAA-3') from the gDNA of strain B1 of *Leuconsotoc mesenteroides* in a 2720 Thermal cycler (Life Technologies, United States) with the following program: 95°C for 10 min, then 40 cycles of 95°C 30 s, 59°C 30 s, 72°C 30 s and a final amplification of 7 min at 72°C.

The amount of the different plasmids were quantified using a NanoDrop One (Thermofisher Scientific, United States) and the number of copies per microliter was calculated as stated by Berthoud et al. [34]. Standard curves ranging from 1E+08 copies/reaction to 1E+02 copies/reaction were included in all qPCR runs.
