# Supplementary figures and images for: Antilisterial Properties of Selected Strains from the Autochthonous Microbiota of a Swiss Artisan Soft Smear Cheese
Source: Foods. 2024 Oct 30;13(21):3473. doi: 10.3390/foods13213473 (PMC11545730; doi:10.3390/foods13213473)

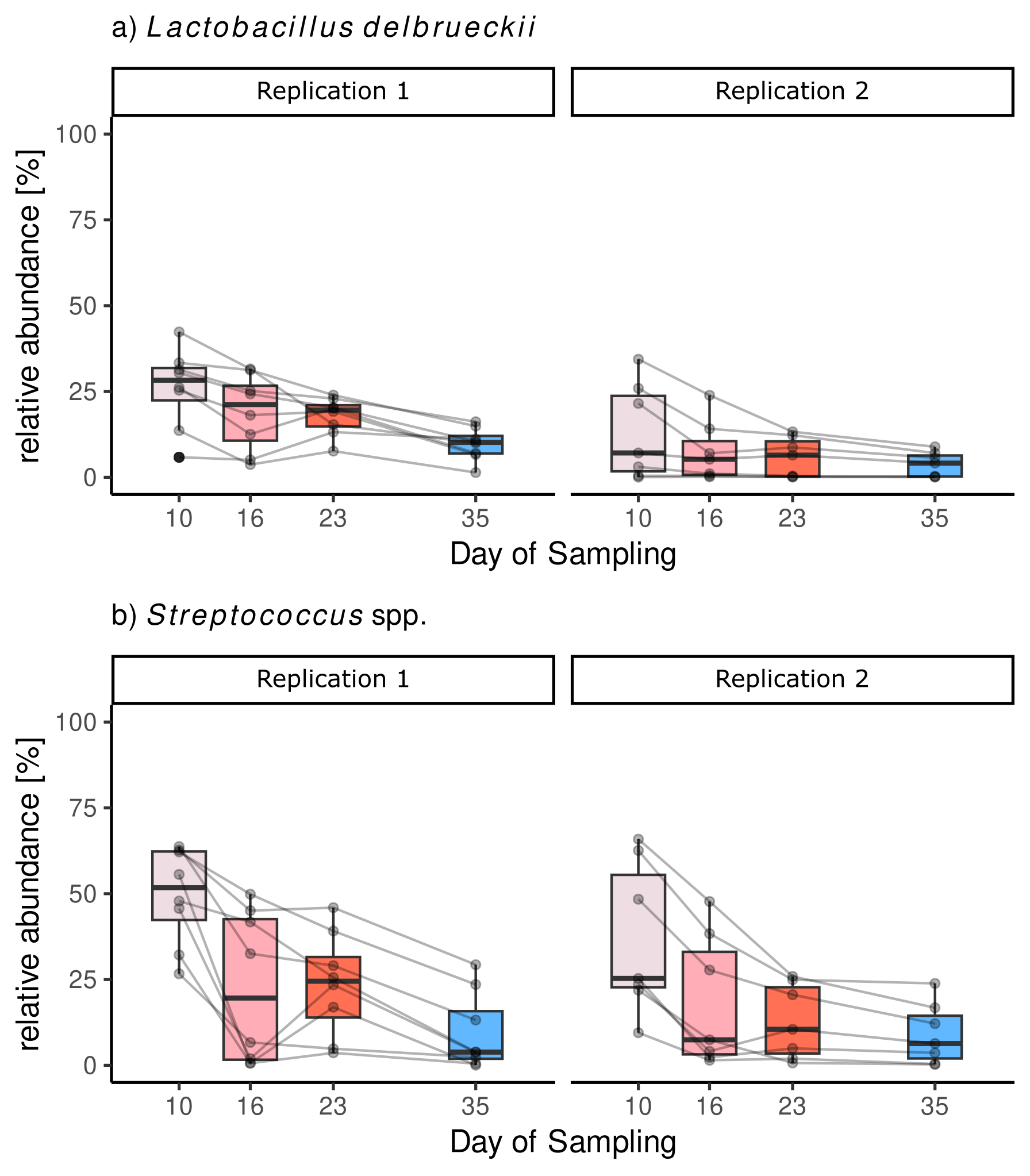

Supplement: Supplementary file 1 [file foods-13-03473-s001.zip › Figure_S1_starter_culture.png]
